# ^125^I Seed Brachytherapy for Refractory Loco-Regional Recurrence of Non-Anaplastic Thyroid Cancer

**DOI:** 10.3389/fonc.2022.773708

**Published:** 2022-02-15

**Authors:** Huimin Yu, Hongtao Zhang, Zhen Gao, Xiaoli Liu, Lijuan Zhang, Xuemin Di, Zeyang Wang, Zezhou Liu, Aixia Sui, Juan Wang, Gaofeng Shi

**Affiliations:** ^1^Department of Oncology, Hebei General Hospital, Shijiazhuang, China; ^2^The 4th Affiliated Hospital of Hebei Medical University, Shijiazhuang, China

**Keywords:** non-anaplastic thyroid cancer, recurrence, brachytherapy, efficacy evaluation, iodine-125 seeds, safety evaluation, refractory

## Abstract

**Purpose:**

The purpose of this research was to evaluate the feasibility and efficacy of ^125^I seed brachytherapy as salvage treatment for recurrence from non-anaplastic thyroid cancer refractory to other modalities.

**Methods:**

Between June 2006 and September 2019, fifteen patients with recurrent non-anaplastic thyroid cancer were treated with ^125^I seed brachytherapy. ^125^I seeds were implanted into the tumor under the guidance of CT and/or ultrasound images with the median prescription dose of 120 Gy (range, 100-140 Gy). The median seed number was 80 (range 10-214). Clinical efficacy was evaluated with Response Evaluation Criteria in Solid Tumors.

**Findings:**

Fifteen patients were selected, eleven of whom had papillary carcinoma, two suffered from follicular carcinoma, and two were diagnosed with medullary carcinoma. These patients had twenty-four nodes in total. After they received salvage surgery and/or radioactive iodine (RAI) therapy, local recurrence was detected in all of them. No less than one node was observed in everyone’s cervical or supraclavicular areas, and four patients had lung metastatic. The median follow-up period lasted 48 months (range, 5-93 months). All patients did not develop locoregional recurrence after experiencing ^125^I seed brachytherapy. Only three of them formed new metastases in nontarget regional nodes after brachytherapy, and additional brachytherapy can solve all regional failure problems. No significant adverse events were observed in any patient.

**Implications:**

For the chosen patients, 125I seed brachytherapy is feasible for treating refractory local recurrence from non-anaplastic thyroid cancer. Further studies are required to determine the role of 125I seed brachytherapy in the treatment of thyroid cancer.

## Introduction

Thyroid cancers are primarily papillary, follicular, medullary, or anaplastic thyroid cancer (1, 2). Surgery remains the mainstay of treatment for non-anaplastic thyroid cancer. The most recommended management of aggressive thyroid cancers is the surgical removal of the thyroid gland (thyroidectomy) followed by radioactive iodine (RI) ablation and thyroid-stimulating hormone (TSH) suppression therapy (3, 4). Early-stage non-anaplastic thyroid cancer is treated with surgery ± RI with recurrence rates of 15–22%. However, recurrence rates for Stage III and IV non-anaplastic thyroid cancer after surgery ± RI are 46% and 66% respectively (5).

Thyroid cancer is insensitive to conventional chemotherapy. Many targeted drugs are increasingly being used to treat refractory loco-regional recurrence of non-anaplastic thyroid cancer due to many signaling pathways and gene mutations that driving thyroid tumorigenesis have been identified (6, 7). Sorafenib and lenvatinib have been approved by the U.S. Food and Drug Administration to be used in radioactive iodine refractory differentiated thyroid cancer,although they have been demonstrated to improve progression free survival and overall response rate, the serious toxicities may affect patients’ quality of life (8, 9).

Loco-regional recurrence in patients with thyroid cancer is recommended to undergo local treatment such as surgical resection, ethanol ablation, radiofrequency ablation and involved-field radiotherapy (10, 11). Resection again is difficult to remove the tumor completely and have a high rate of recurrent laryngeal injury (12). External beam radiotherapy (EBRT) is not routinely used to treat thyroid cancer. At the same time, it is hard to cure the tumor because of the low radiation sensitivity and dose limit of normal tissue. Furthermore, there is no evidence showing that radiotherapy can prolong overall survival (13). The current indications for EBRT in thyroid cancer have been largely derived from retrospective data (14). Stereotactic body radiotherapy (SBRT) can accurately deliver a high dose of conformal radiation to the target area with a small dose. However, existingresearch reported only nine cases with the largest tumor volume of 43.6ml (15). Therefore, there are insufficient instructions on the role of radiotherapy in thyroid cancer.

The therapeutic research emphasized the importance of individualizing treatment strategies in patients with a recurrence of disease limited to a local site (4). As brachytherapy, ^198^Au, ^192^Ir and ^125^I seed were reported to treat recurrent thyroid cancer and got effective results (16–18). In this context, we propose a valuable salvage technique using image-guided ^125^I seed brachytherapy to treat the refractory recurrence of non-anaplastic thyroid cancer after conventional treatment.

## Methods and Materials

### Clinical Information

Our hospital indications for ^125^I seed brachytherapy are as follows: 1) non-anaplastic thyroid cancer; 2) recurrent LNs stated inoperable or unresectable by experienced head and neck surgeons; 3) LN recurrence refractory to repeated RAI treatment; and 4) unfit for resection and RAI reviewed by multidisciplinary tumor board; 5) a Karnofsky Performance Score (KPS) of 70 or higher. The exclusion criteria applied are thyroid bed recurrence, any single LN greater than 10 cm, more than five metastatic LNs, major organ dysfunction, acute or chronic infections, severe organ and coagulation dysfunction, lethal important organ metastases. In all cases, whether delivering brachytherapy or not will be discussed by our multidisciplinary tumor board. Once the treatment is recommended, we will inform the patients of benefits and potential harm of brachytherapy. Then patients are required to submit informed written consent before participating in the protocol. Having received approval from the Institutional Review Board, we reviewed the medical records of fifteen consecutive patients who were treated by brachytherapy for LN recurrence of non-anaplastic thyroid cancer from June 2006 to September 2019 at our institution. Patients and tumor characteristics have been summarized in **Table 1**.

**Table 1:** History of previous treatment in fifteen patients who received brachytherapy for neck LN recurrence.

	Sex	Age (year)	Diagnosis	Surgery location	Primary/retreatment	Total LN volume (cm3)*	Seed activity (mCi)	Prescription dose (Gy)
1	Male	54	PTC	l-NLN, r-NLN	9 neck surgeries	107.5	0.4/0.6	120
					3 RI treatments			
2	Female	56	PTC	r-NLN	2 neck surgeries	22.5	0.4	120
					1 RI treatments			
3	Female	69	FTC	l-NLN	5 neck surgeries	12	0.5	100
4	Male	64	FTC	l-MLN, dorsal metastasis	1 neck surgery	448	0.5-0.7	100
					1 endotracheal stent implant			
5	Male	53	MTC	l-NLN, l-NLN	1 neck surgery	140	0.3/0.4	120
					1 chemotherapy			
					1 neck EBRT			
6	Male	46	PTC	r-NLN, l-NLN, Lung Metastasis	2 neck surgeries	22.4	0.3	100/140
					1 neck EBRT			
7	Male	53	PTC	l-NLN, r-NLN	4 neck surgeries	85.2	0.5	100
					1 RI treatment			
					1 125I seeds			
8	Male	46	PTC	l-NLN, l-NLN	1 neck surgery	8.2	0.3/0.4	100
					3RI treatments			
9	Male	47	PTC	l-NLN	2 neck surgeries	165.5	0.3-3.0	140
10	Male	68	PTC	r-NLN, l-NLN, Lung Metastasis	3 neck surgeries	61	0.5	120
					4 RI treatments			
11	Female	54	PTC	l-NLN	3 neck surgeries	7.2	0.5	120
					1 RI treatment			
12	Male	78	PTC	Submental lymph node	3 neck surgeries	15.4	0.3	120
					3RI treatment			
					1 neck EBRT			
13	Female	63	PTC	l-NLN	1 neck surgery	60	0.6	120
14	Female	58	PTC	l-NLN	1 neck surgery	100	0.65	120
					1 chemotherapy			
					1 brain SBRT			
15	Female	67	MTC	l-NLN	2 neck surgeries	30	0.6	120
					1 mediastinum EBRT			

PTC, papillary thyroid cancer; FTC, follicular thyroid cancer; MTC, medullary thyroid cancer; l-NLN, left neck lymph node; RI, radioactive iodine; r-NLN, right neck lymph node; l-MLN, left-mediastinum lymph node; SBRT, stereotactic body radiotherapy; EBRT, external beam radiotherapy.

*Total LN volume is a sum of volumes of all treated LNs.

### Treatment

#### Preoperative Planning

The patients were immobilized with the vacuum cushion in the treatment position. A position line was drawn using the CT laser on the surface of the patient’s skin around the tumor location, and two marks were made 3–4 cm away on this line. Contrast-enhanced CT with a 5-mm slice thickness was performed. The planning was performed using the Prowess treatment planning system (TPS) (Panther Brachy version 5.0 TPS, Prowess Inc., Concord, CA, USA). The gross target volume (GTV) was defined as metastatic LN visible on CT images. Clinical target volume (CTV) was generated by expanding GTV by 3 mm three-dimensionally. The ultrasound images used in the metastatic LNs are invisible on CT images. Then the seed activity was selected, the needles were designed, and seeds were loaded. The prescription dose was 100 to 140 Gy. The pre-plan was committed when the target dose approached the prescription dose while normal tissue doses were minimized. If the seeds were implanted under the guidance of 3D-printed template, four steps were necessary: 1) the CT image series and space coordinates of all the needle locations were then exported after pre-plan; 2) the patient’s area of skin, the template to be printed, and needle coordinates were reconstructed; 3) the 3D-printed templates were printed by a 3D printer (Unicorn 3DSL450M, Beijing Unicorn Science and Technology Ltd., Beijing, China); 4) 1 day before operation, the 3D−printed template was disinfected and sterilized.

#### Seed Activity Measurement

A radioactivity meter (RM−905a well−type ionization chamber, National Institute of Metrology, BeiJing, China) was used to measure the activity of radioactive ^125^I seeds (model, 6711−99; activity, 0.3–3.0 mCi; length, 4.5 mm; diameter, 0.8 mm; average energy, 27–35 keV; Beijing Zhibo Pharmaceutical Company, BeiJing, China) before the operation. If activity error was <5%, all seeds would be blocked in the shell of brachytherapy applicator for disinfection.

#### Brachytherapy Procedure

During the operation, the vacuum cushion was used to fix the patient into position, ensuring the position was the same as that during the preoperative planning. The proper CT laser position was ensured such that the laser line aligns with the line drawn on the patient’s skin surface preoperatively. The needles were inserted into the tumor guided by a CT scan using a freehand implantation technique. If the seeds were implanted guided by a 3D−printed template, the 3D−printed template was disinfected with formaldehyde through a fumigation process and then was secured on the patient’s body surface based on the markers attached. A CT scan was performed to confirm that the template location was correct, and then the needles were fed into the tumor target based on the position of the template holes. After inserting all the needles, a CT scan was performed again to confirm the needle positions. Finally, the radioactive seeds were implanted according to a preoperative plan. If the tumor was invisible on the CT image, the seeds were implanted under the guidance of ultrasound.

### Postoperative Verification Plan

After the procedure, another CT scan was performed immediately, with a slice thickness of 5 mm. The images were then transferred into the TPS to verify the dose distribution. The TPS was used to contour the target volume and the OARs, as well as identify the implanted seeds. Subsequently, the isodose lines and D90, seed number, and DVH were determined.

### Follow-Up and Evaluation

Follow-up CT images and/or ultrasound images were used to investigate the responses of recurrent LNs to brachytherapy. Complete response (CR) was defined as the complete disappearance of the target. Partial response (PR) referred to at least a 50 percent decrease in the short axis of the target. And progressive disease (PD) meant at least a 20 percent increase in the short axis of the target. When there was not enough contraction to meet PR or insufficient growth to reach PD, people had stable disease (SD). In accordance with the National Cancer Institute Common Toxicity Criteria v4.0, we will also score the morbidity after brachytherapy.

### Statistical Analysis

The prescription dose for SBRT was converted to an equivalent dose in 2-Gy fractions (EQD2). In a case where a daily fraction dose of *d* (Gy) was delivered in *n* fractions, EQD2 (Gy) was calculated as follows: $\text{EQD}2=D\left[1+\frac{R0}{\left(\mu +\lambda )(\frac{\alpha }{\beta }\right)}\right]/\left[1+\frac{2}{\frac{\alpha }{\beta }}\right]$ . D is the D90 of brachytherapy, *R_0_* is the initial dose rate, μ is the cell repair constant equals to 0.5h^-1^. λ is the seed decay constant equals to lin2/T_1/2_, α/β ratio of 10 was used to obtain the EQD2 for the tumor. The Kaplan-Meier method was used to estimate survival rates. Follow-up was timed from the first brachytherapy procedure. All statistical tests were performed using the 13.0 version SPSS statistical software (IBM, Armonk, NY USA).

## Results

### Patient Features and Pre-Brachytherapy Remedy

Between June 2006 and September 2019, fifteen patients were enrolled in the study and received brachytherapy. Among these patients, 60% were male and 40% were female. Their median age was 56 years (range 46-78 yrs). KPSs ranged from 80 to 100. Eleven patients had papillary carcinoma, two had follicular carcinoma, and two had medullary carcinoma. Synchronous distant metastasis was observed in five patients when implementing brachytherapy.Three had metastatic lesions in the lung, one patient had brain metastasis treated by SBRT and one had pleura metastases. All patients received at least one surgery and patient 1 received nine surgeries. Seven patients received RI treatment. Two patients were treated by chemotherapy and four patients were treated by EBRT. One patient with brain metastasis was treated by brain SBRT (γ-knife). One patient with tracheostenosis received endotracheal stent implantation. One patient was implanted ^125^I seeds with a very low prescribed dose. All patients had LN metastases in their neck by CT and/or ultrasound scans. Before receiving brachytherapy, these patients had developed thyroid cancer recurrence, distantly or in the neck. Patient characteristics are summarized in **Table 1**.

### Brachytherapy Characteristics

In fifteen patients, a total of 24 metastatic LNs were treated in 36 brachytherapy sessions. Brachytherapy delivery and results are detailed in **Table 2**. The number of LNs treated per brachytherapy session ranged from one to two. Brachytherapy was administered more than once to most patients except six patients (patients 2, 11, 12, 13, 14, and 15). One patient experienced five sessions of brachytherapy in a year (patient 9). In their first brachytherapy sessions, all patients received brachytherapy directed to cervical LN metastases. Nevertheless, for most patients, it is difficult to give the LN an adequate dose in one session of brachytherapy. Brachytherapy was administered using the freehand technique in nine patients and a 3D-printed template technique in 6 patients. The median isodose level was 100 percent (range 90%-131.7%).

**Table 2:** Details and outcomes of brachytherapy in fifteen patients.

Patient no.	Brachytherapy session	LN (s) (n)	Seeds number	Brachytherapy dose (Gy)	Implant and guide technique	Brachytherapy EQD2 (Gy)	Response	Status (mo)
1	4	2	150	120	FH/CT and US	102	CR	DWD(48)
2	1	1	31	110	FH/CT and US	93.5	CR	NED(92)
3	2	1	89	95/110	FH/CT and US	80.75/93.5	CR/SD	DWD(93)
4	5	2	214	90	FH/CT	76.5	PR	DWD(59)
5	3	2	90	110	FH/CT and US	93.5	CR	AWD(64)
6	5	3	89	100/140	FH/CT and US	85/119	CR	AWD(56)
7	2	2	83	107/100	3D/CT	90.95/85	SD	DWD(31)
8	2	2	22	131.7	FH/US	111.9	CR	AWD(52)
9	5	1	179	145	3D/CT	123.25	PR	AWD(27)
10	2	3	52	123/124	3D/CT	104.55/105.4	PR	DWD(65)
11	1	1	10	120	FH/CT and US	102	CR	NED (21)
12	1	1	41	118	3D/CT	100.3	PR	AWD(5)
13	1	1	70	121	FH/CT and US	102.85	CR	DWD(15)
14	1	1	60	120	FH/CT and US	102	CR	AWD(12)
15	1	1	80	120	FH/CT and US	102	PR	DWD(23)

AWD, alive with disease; DWD, dead with disease; NED, no evidence of disease; EQD2, equivalent dose in 2-Gy fraction; 3D, 3D-printed template guided brachytherapy; FH, Freehand brachytherapy; US, ultrasound image.

### Treatment Outcomes

**Figure 1:**
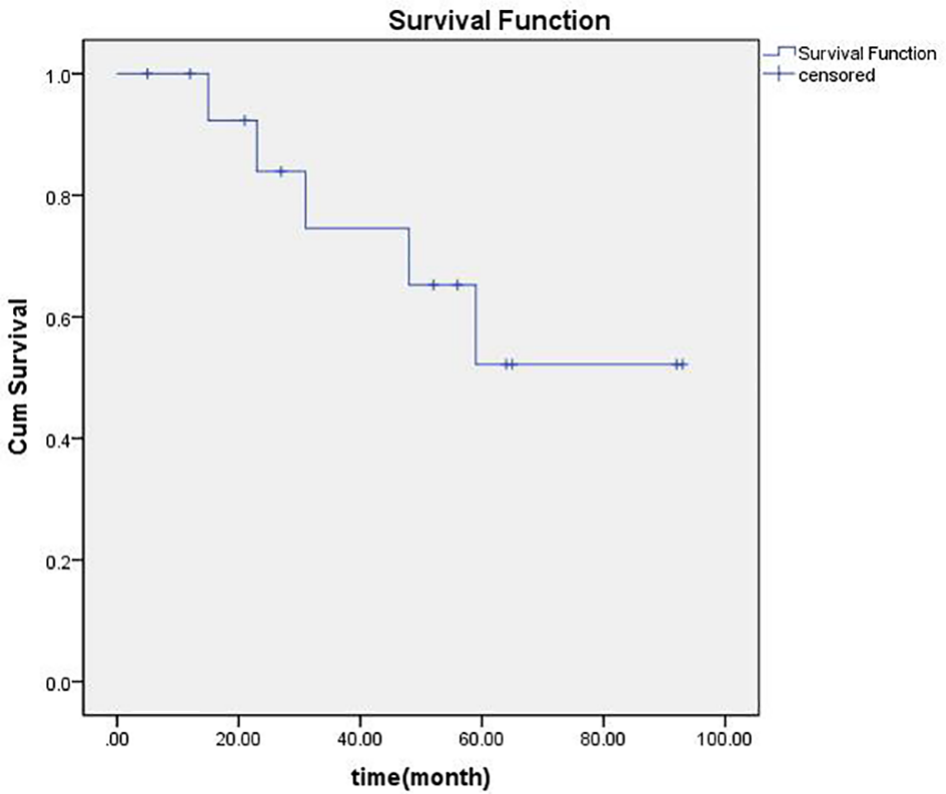
The overall survival rates of the patients after ^125^I seed brachytherapy.

Median follow-up was 48 months ranging from 5 to93 months. There was no local PD for any of the 24 LNs treated with brachytherapy. The number of LNs with CR, PR, and SD were respectively fifteen, six, and three. Regional failures were observed in four patients: one with papillary carcinoma (patients 10), one with medullary carcinoma (patient 5), and two with follicular thyroid cancer (patients 3 and 4). The 1-, 2-, 3-, 4-, and 5-year overall locoregional control rate was 92.9%, 83.3%, 54.6%, 45.5%, and 40%, respectively, and the 1-, 2-, 3-, 4-, and 5-year overall survival rate was, respectively, 91%, 82%, 71%, 58%, and 41%. An actuarial curve for overall survival is illustrated in **Figure 1**. Of these, two patients (patients 3 and 10) received additional sessions of brachytherapy directed to newly developed metastatic LNs. After additional sessions of brachytherapy for regional failure, one patient developed no further neck metastases and was rendered free of neck disease, the other died three months after the procedure because of heart failure. Three patients with lung metastases before the first brachytherapy procedure. One of them (patient 8) was ablated by microwave, one (patient 12) was told to receive active surveillance, and the other (patient 6) was treated by brachytherapy. Six patients developed distant metastases during follow-up. One (patient 1) developed brain metastasis, two (patients 3 and 4) lung metastasis, one (patient 7) mediastinum lymph node metastasis, one (patient 5) bone metastasis, and the other (patient 10) brain, lung and bone. Compared with brachytherapy, no grade three or higher adverse reactions were observed.

## Discussion

It is well known that thyroid cancer has innocent biological behavior and most of the patients have excellent prognosis. However, Zhu’s study showed an increasing incidence of recurrent thyroid cancer recently (19). Once recurrent, some locoregional treatment methods such as radiotherapy, surgery, radiofrequency ablation and brachytherapy can be used to treat recurrent thyroid cancer (11, 20–22). Butfor refractory recurrence of thyroid cancer, there is still no optimal treatment. Terezakis SA reported results for adjuvant and salvage EBRT in 76 patients with non-anaplastic thyroid cancer, and in patients with macroscopic residual disease, the four-year locoregional control rate was 62 percent (23). Kim et al. studied stereotactic body radiotherapy as salvage treatment for cervical recurrence from non-anaplastic thyroid cancer refractory to other modalities. No local progression was observed in the nodes during the 23-month follow-up (15). Parker et al. treated a patient with cerebral metastasis by craniotomy, postoperative external cerebral radiotherapy and ^125^I seed implantation in the tumor bed and made a complete recovery (24). This study was the pioneer of using ^125^I to treat cerebral metastasis from thyroid cancer. However, this paper studied only tumor bed implantation for cerebral metastasis after surgery. From 1990, no other report about ^125^I seed implantation used in recurrent thyroid cancer was found in PubMed. ^125^I seed implantation is a brachytherapy that belongs to radiotherapy. Numerous studies on outcomes of EBRT for thyroid cancer used elective nodal irradiation ENI (25–27). However, ENI is related to lengthy duration of treatment and radiation-induced morbidity. In addition, it remains to be determined whether ENI can decrease the possibilities of local recurrence in patients with recurrent thyroid cancerand ultimately improve relapse-free rates remains to be ascertained (28). In Terezakis’s study, the median total EBRT dose delivered was 63 Gy (59.4 – 70Gy). The overall local control rate was 77% for two years and 62% for four years for those with gross residual disease, and the 2- and 4-year overall survival rates were respectively 57% and 46% for those with gross residual disease (23). In Kim’s study, the dose (EQD2) delivered was 50 - 74.7Gy, and the overall survival rate was not reported. Five of nine patients developed regional failures, and the 1-year local control rate was 78% (15). In our study, the dose (EQD2) delivered was 85-123.25Gy and the overall local control rate was 92.9% for one year, 83.3% for two years, and 45.5%% for four years, the 2- and 4-year overall survival rate for all patients was 82% and 58%. Our 1-year, 2-year local control rate and 2-year, 4-year survival rate were better than Kim’s and Terezakis’s study respectively. The reason may have stemmed from our higher dose delivered. According to Kim’s study, SBRT seems only to be used for small recurrent thyroid cancer (1.6ml to 43.6ml) (15), while ^125^I seed brachytherapy can treat thyroid cancer with a larger volume (7.2ml to 448ml). Our 4-year local control rate is lower than EBRT. Two reasons may need to be taken into account. The first is the targets were the tumor bed with gross residual disease after surgery in the EBRT group while the targets were recurrent tumors in our study. The second is the treatment volume in the EBRT group may be much bigger than our study. Ji et al. reported a 44.2% 5-year local control rate if D90 was ≥120 Gy for recurrent head and neck tumors after radiation therapy treated by 125I seed brachytherapy (29). Our 5-year locoregional control rate (40%) is a little lower compared to Ji’s study. It may be mainly because the D90 of some patients in our study was not adequate. Compared to EBRT, the most important advantage of ^125^I seed brachytherapy is improving the dose of the target and at the same time, lowering the dose of the organ at risk. If we elevate the D90 of the target and at the same time control the dose of organ at risk, the results might be better. Furthermore, multiple times of salvage ^125^I seed treatment after local failure is another advantage of brachytherapy.

For ENI, the main disadvantage of treating thyroid cancer is radiation-related toxicity. The most common adverse reactions are dysphagia, pharyngitis, hoarseness, mucositis,dermatitis, and some late complications, such as tracheal stenosis, skin fibrosis, and esophageal stricture. One study reported that among 76 patients who underwent EBRT, 24 of them suffered from grade 3 dysphagia and 14 of them mucositis (23). Another study of 131 patients treated with EBRT asserted that ten patients had severe late radiation-induced morbidities; required dilation for esophageal stricture occurred in seven patients and two developed tracheal stenosis (30). However, there were no radiation-related toxicities in the present study, implying that ^125^I seed brachytherapy with a dose (EQD2) of 85-123.25Gy was safe for previously treated patients No brachytherapy related morbidities were observed during the up to 93 months follow-up. This may owe to the strict dose control of the organ at risk.

## Conclusions

^125^I seed brachytherapy was effective for controlling LN recurrence in thyroid cancer. Compared to EBRT and SBRT, brachytherapy has better local control, overall survival, and lower radiation-induced complications. Nevertheless, further large-scale research should be carried out to determine the role of brachytherapy in patients with local recurrence of thyroid cancer.

## Data Availability Statement

The original contributions presented in the study are included in the article/supplementary material. Further inquiries can be directed to the corresponding author.

## Ethics Statement

The studies involving human participants were reviewed and approved by the ethics committee of Hebei Provincial People’s Hospital. The patients/participants provided their written informed consent to participate in this study.

## Author Contributions

All authors listed have made a substantial, direct, and intellectual contribution to the work and approved it for publication.

## Conflict of Interest

The authors declare that the research was conducted in the absence of any commercial or financial relationships that could be construed as a potential conflict of interest.

## Publisher’s Note

All claims expressed in this article are solely those of the authors and do not necessarily represent those of their affiliated organizations, or those of the publisher, the editors and the reviewers. Any product that may be evaluated in this article, or claim that may be made by its manufacturer, is not guaranteed or endorsed by the publisher.

## References

[1] Brose MS Nutting CM Jarzab B Elisei R Siena S Bastholt L. Sorafenib in Radioactive Iodine-Refractory, Locally Advanced or Metastatic Differentiated Thyroid Cancer: A Randomised, Double-Blind, Phase 3 Trial. Lancet (2014) 384(9940):319–28. doi: 10.1016/S0140-6736(14)60421-9 PMC4366116 24768112

[2] Schlumberger M Sherman S. ENDOCRINE TUMOURS: Approach to the Patient With Advanced Differentiated Thyroid Cancer. Eur J Endocrinol (2012) 166(1):5–11. doi: 10.1530/EJE-11-0631 21890651

[3] Nguyen QT Lee EJ Huang MG Park YI Plodkowski RA. Diagnosis and Treatment of Patients With Thyroid Cancer. Am Health Drug Benefits (2015) 8(1):30. PMC4415174 25964831

[4] Jillard CL Scheri RP Sosa JA. What Is the Optimal Treatment of Papillary Thyroid Cancer? Adv Surg (2015) 49(1):79–93. doi: 10.1016/j.yasu.2015.03.007 26299491

[5] Loh KC Greenspan FS Gee L Miller TR Yeo P. Pathological Tumor-Node-Metastasis (pTNM) Staging for Papillary and Follicular Thyroid Carcinomas: A Retrospective Analysis of 700 Patients. J Clin Endocrinol Metab (1997) 82(11):3553–62. doi: 10.1210/jcem.82.11.4373 9360506

[6] Lin YS Yang H Ding Y Cheng YZ Shi F Tan J. Donafenib in Progressive Locally Advanced or Metastatic Radioactive Iodine-Refractory Differentiated Thyroid Cancer: Results of a Randomized, Multicenter Phase II Trial. Thyroid (2020) 31(4):607–15. doi: 10.1089/thy.2020.0235 32907500

[7] Bible KC Menefee ME Lin CJ Millward MJ Erlichman C. An International Phase 2 Study of Pazopanib in Progressive and Metastatic Thyroglobulin Antibody Negative Radioactive Iodine Refractory Differentiated Thyroid Cancer. Thyroid (2020) 30:1254–62. doi: 10.1089/thy.2019.0269 PMC7482116 32538690

[8] Costante G. Multikinase Inhibitors for the Treatment of Radioiodine Refractory Thyroid Cancer: What Have We Learned From the 'Real-World' Experience? Curr Opin Oncol (2021) 33(1):3–8. doi: 10.1097/CCO.0000000000000693 33060402

[9] Feola T Cozzolino A Centello R Pandozzi C Tarsitano M Giannetta E. Predictors of Response and Survival to Multikinase Inhibitors in Radioiodine Resistant Differentiated Thyroid Cancer. J Pers Med (2021) 11(7):674. doi: 10.3390/jpm11070674 34357141 PMC8306329

[10] Haugen BR Alexander EK Bible KC Doherty G Mandel SJ Nikiforov YE. 2015 American Thyroid Association Management Guidelines for Adult Patients With Thyroid Nodules and Differentiated Thyroid Cancer: The American Thyroid Association Guidelines Task Force on Thyroid Nodules and Differentiated Thyroid Cancer. Thyroid (2016) 26(1):1–133. doi: 10.1089/thy.2015.0020 26462967 PMC4739132

[11] Fugazzola L Elisei R Fuhrer D Jarzab B Leboulleux S Newbold K. 2019 European Thyroid Association Guidelines for the Treatment and Follow-Up of Advanced Radioiodine-Refractory Thyroid Cancer. Eur Thyroid J (2019) 8(5):227–45. doi: 10.1159/000502229 PMC6873012 31768334

[12] Roh J-L Kim J-M Park CI. Central Compartment Reoperation for Recurrent/Persistent Differentiated Thyroid Cancer: Patterns of Recurrence, Morbidity, and Prediction of Postoperative Hypocalcemia. Ann Surg Oncol (2011) 18(5):1312–8. doi: 10.1245/s10434-010-1470-9 21140230

[13] Durante C Haddy N Baudin E Leboulleux S Hartl D Travagli JP. Long-Term Outcome of 444 Patients With Distant Metastases From Papillary and Follicular Thyroid Carcinoma: Benefits and Limits of Radioiodine Therapy. J Clin Endocrinol Metab (2006) 91(8):2892–9. doi: 10.1210/jc.2005-2838 16684830

[14] Brierley JD Tsang RW. External Beam Radiation Therapy for Thyroid Cancer. Endocrinol Metab Clin North Am (2008) 37(2):497–509. doi: 10.1016/j.ecl.2008.02.001 18502339

[15] Kim JH Kim M-S Yoo SY Lim SM Lee GH Yi KH. Stereotactic Body Radiotherapy for Refractory Cervical Lymph Node Recurrence of Nonanaplastic Thyroid Cancer. Otolaryngology—Head Neck Surg (2010) 142(3):338–43. doi: 10.1016/j.otohns.2009.12.034 20172377

[16] Rozdilsky S Tkacenko G Vasiljev L Nacinova NA. Interstitial Radiotherapy of Thyroid Cancer Using 198au-Comizol. Radiobiol Radiother (Berl) (1988) 29(6):729. 3253796

[17] Wu N Zhao H Han D Cheng G Zhao Z Ge Y. Image-Guided High-Dose-Rate Interstitial Brachytherapy–A Valuable Salvage Treatment Approach for Loco-Regional Recurrence of Papillary Thyroid Cancer. J Contemp Brachytherapy (2016) 2(2):150–5. doi: 10.5114/jcb.2016.59127 PMC4873548 27257420

[18] Kanitz W Kopp J Hamperl W Hei De Nreich P Wagner T. Interstitial Radiotherapy With 125i Seeds in Non-Operable and Non-Radioiodine Retaining Local Recurrences of Differentiated and Undifferentiated Thyroid Cancers. Wiener Klin Wochenschr (1990) 102(9):277–80. 2375118

[19] Zhu J Wang X Zhang X Li P Hou H. Clinicopathological Features of Recurrent Papillary Thyroid Cancer. Diagn Pathol (2015) 10(1):96. doi: 10.1186/s13000-015-0346-5 26168921 PMC4501206

[20] Grant CS. Recurrence of Papillary Thyroid Cancer After Optimized Surgery. Gland Surg (2015) 4(1):52. doi: 10.3978/j.issn.2227-684X.2014.12.06 25713780 PMC4321046

[21] Meadows KM Amdur RJ Morris CG Villaret DB Mazzaferri EL Mendenhallet WM. External Beam Radiotherapy for Differentiated Thyroid Cancer. Am J Otolaryngol (2006) 27(1):24–8. doi: 10.1016/j.amjoto.2005.05.017 16360819

[22] Wolf G Kohek P Geyer E Pakisch B Passath A. Intraoperative Radiation Therapy, Endotracheal Hyperthermia and IR-192-Brachytherapy in Patients With Advanced Thyroid Cancer. Acta Med Austriaca (1996) 23(1-2):76. doi: 10.1001/jama.275.1.70 8767520

[23] Terezakis SA Lee KS Ghossein RA Rivera M Tuttle RM Wolden SL. Role of External Beam Radiotherapy in Patients With Advanced or Recurrent Nonanaplastic Thyroid Cancer: Memorial Sloan-Kettering Cancer Center Experience. J Radiat Oncol Biol Phys (2009) 73(3):795–801. doi: 10.1016/j.ijrobp.2008.05.012 18676097

[24] Parker LN Wu S-Y Kim DD. Recurrence of Papillary Thyroid Carcinoma Presenting as a Focal Neurologic Deficit. Arch Intern Med (1986) 146(10):1985. doi: 10.1001/archinte.1986.00360220145025 3767543

[25] Jamshid FMD Christoph RMD Martin SMB Stefan PMMD Horst SMD. Differentiated Thyroid Cancer: Impact of Adjuvant External Radiotherapy in Patients With Perithyroidal Tumor Infiltration (Stage Pt4). Cancer (1996) 77(1):172–80. doi: 10.1002/(SICI)1097-0142(19960101)77:13.0.CO;2-1 8630926

[26] Keum KC Suh YG Koom WS Cho JH Su JS Chang GL. The Role of Postoperative External-Beam Radiotherapy in the Management of Patients With Papillary Thyroid Cancer Invading the Trachea. Int J Radiat Oncol Biol Phys (2006) 65(2):474–80. doi: 10.1016/j.ijrobp.2005.12.010 16542796

[27] Kim TH Yang D-S Jung K-Y Kim CY Choi MS. Value of External Irradiation for Locally Advanced Papillary Thyroid Cancer. Int J Radiat Oncol Biol Phys (2003) 55(4):1006–12. doi: 10.1016/S0360-3016(02)04203-7 12605980

[28] Lee N Tuttle M. The Role of External Beam Radiotherapy in the Treatment of Papillary Thyroid Cancer. Endocr Relat Cancer (2006) 13(4):971–7. doi: 10.1677/ERC-06-0039 17158749

[29] Ji Z Jiang Y Tian S Guo F Peng R Xu F. The Effectiveness and Prognostic Factors of CT-Guided Radioactive I-125 Seed Implantation for the Treatment of Recurrent Head and Neck Cancer After External Beam Radiation Therapy. Int J Radiat Oncol Biol Phys (2019) 103(3):638–45. doi: 10.1016/j.ijrobp.2018.10.034 30391521

[30] Schwartz DL Lobo MJ Ang KK Morrison WH Rosenthal DI Ahamad A. Postoperative External Beam Radiotherapy for Differentiated Thyroid Cancer: Outcomes and Morbidity With Conformal Treatment. Int J Radiat Oncol Biol Phys (2009) 74(4):1083–91. doi: 10.1016/j.ijrobp.2008.09.023 PMC2745400 19095376

